# A Berberine Bridge Enzyme-Like Protein, *GmBBE-like43*, Confers Soybean's Coordinated Adaptation to Aluminum Toxicity and Phosphorus Deficiency

**DOI:** 10.3389/fpls.2022.947986

**Published:** 2022-08-08

**Authors:** Qianqian Chen, Jifu Li, Guoxuan Liu, Xing Lu, Kang Chen, Jiang Tian, Cuiyue Liang

**Affiliations:** State Key Laboratory for Conservation and Utilization of Subtropical Agro-Bioresources, Root Biology Center, South China Agricultural University, Guangzhou, China

**Keywords:** soybean, berberine bridge enzyme-like protein, phosphorus deficiency, aluminum toxicity, oligogalacturonides

## Abstract

Phosphorus (P) deficiency and aluminum (Al) toxicity often coexist and are two major limiting factors for crop production in acid soils. The purpose of this study was to characterize the function of *GmBBE-like43*, a berberine bridge enzyme-like protein-encoding gene, in soybean (*Glycine max*) adaptation to Al and low P stresses. Present quantitative real-time PCR (qRT-PCR) assays confirmed the phosphate (Pi)-starvation enhanced and Al-stress up-regulated expression pattern of *GmBBE-like43* in soybean roots. Meanwhile, the expression of a *GmBBE-like43-GFP* chimera in both common bean hairy roots and tobacco leaves demonstrated its cell wall localization. Moreover, both transgenic Arabidopsis and soybean hairy roots revealed the function of *GmBBE-like43* in promoting root growth under both Al and low P stresses. *GmBBE-like43*-overexpression also resulted in more H_2_O_2_ production on transgenic soybean hairy root surface with oligogalacturonides (OGs) application and antagonized the effects of Al on the expression of two *SAUR-like* genes. Taken together, our results suggest that *GmBBE-like43* might be involved in the soybean's coordinated adaptation to Al toxicity and Pi starvation through modulation of OGs-oxidation in the cell wall.

## Introduction

Aluminum toxicity and low Pi availability are two major factors limiting crop production on acid soils in tropic and sub-tropic areas (Kochian et al., 2004). Due to the coexistence of Al toxicity and P deficiency in acid soils, understanding how plants coordinately adapt to Al-P couple stresses are important for crops' genetic improvement. To date, two strategies underlying P-improved plant Al resistance have been suggested. One is based on the immobilization of Al by Pi in the root cell wall, which has been reported in buckwheat (*Fagopyrum esculentum*), barley (*Hordeum vulgare*), and maize (*Zea mays*) (McCormick and Borden, 1972; Gaume et al., 2001; Zheng et al., 2005). The other relied on the activation of organic anion's exudation induced by P application, which was observed in rapa (*Brassica campestris*) and soybean (*Glycine max*) (Liao et al., 2006; Ligaba et al., 2007; Liang et al., 2013). On the other hand, identification and functional characterization of the sensitivity to proton rhizotoxicity 1 (STOP1) zinc-finger transcription factor family have largely expanded the understanding of the regulatory mechanisms of plant adaptation to Pi starvation and Al toxicity (Sawaki et al., 2009; Kobayashi et al., 2014; Gutiérrez-Alanís et al., 2018; Wu et al., 2018a; Wang et al., 2019).

The cell wall is the first barrier of a cell that has direct contact with the environment and plays an essential role in cell division, enlargement, and differentiation. It was suggested that Al toxicity and P deficiency might affect cell wall extension partially by modifying the pectin methylation level (Yang et al., 2008, 2011, 2013; Fernandes et al., 2016). But pectin demethylesterification subsequently increases the generation of active partially de-methylated oligogalacturonides (OGs) (Osorio et al., 2008; Wolf et al., 2012). It thus indicates that Al toxicity and P deficiency could result in increased OGs production, which has been characterized as the plant's damage-associated molecular patterns (DAMPs) and known to influence plant growth and development in several plant species (Simpson et al., 1998; Ferrari et al., 2013). However, prolonged exposure to OGs can also show deleterious outcomes resulting in reduced plant growth (Cervone et al., 1987). One possible reason is that OGs could antagonize the physiological response of the plant to auxin (Branca et al., 1988; Bellincampi et al., 1993; Altamura et al., 1998; Savatin et al., 2011). In Arabidopsis (*Arabidopsis thaliana*), the transcript accumulation of several auxin-induced genes, as well as the activation of the synthetic auxin-responsive promoter DR5, are inhibited by the OGs independently of AtrbohD-mediated H_2_O_2_ accumulation (Savatin et al., 2011; Ferrari et al., 2013). Thus, maintaining the homeostasis of OGs in the cell wall would be important for plant growth under abiotic stresses such as Al toxicity and P deficiency.

The berberine bridge enzyme-like (BBE-like) is a subgroup of the superfamily of FAD-linked oxidases, which feature an unusual bi-covalent attachment of the flavin cofactor *via* His and Cys residues (Daniel et al., 2017). Although plenty of genes encoding BBE-like enzymes have been determined in plants over the course of genome sequencing efforts, the biochemical function of most of them remained elusive (Wallner et al., 2013; Daniel et al., 2017). In Arabidopsis, a total of 28 BBE-like genes were identified. Microarray data demonstrated the high expression of AtBBE-like genes at different developmental stages of plants, especially in root growth, such as lateral root initiation, root elongation and maturation, and proliferation as well as embryonal development (Winter et al., 2007; Daniel et al., 2015, 2016; Wu et al., 2019). Four of them have been identified as OG oxidases (OGOX), including At1g01980 (AtBBE-like1), At1g11770 (AtBBE-like2), At4g20830 (AtBBE-like19 and AtBBE-like20), and At4g20840 (AtBBE-like21) (Daniel et al., 2015; Benedetti et al., 2018). Likewise, CELLOX (At4g20860, AtBBE-like22) was identified as cellodextrin oxidase, which specifically oxidizes cellodextrins with a preference for cellotriose (Locci et al., 2019). According to their structural characterization, AtBBE-like15 and fourteen other AtBBE-like members were speculated to participate in cell wall metabolisms (Daniel et al., 2015). In other plant species, such as citrus fruit and poplar tree, BBE-like homologs were documented to be highly up-regulated in response to osmotic stress and pathogen attack, indicating the functional diversity of plant BBE-like homologs (Attila et al., 2008; González-Candelas et al., 2010).

Previous studies have identified a cell wall located in soybean BBE-like protein GmBBE-like43 (*Glyma.15G134300*), which showed enhanced protein accumulation under Pi starvation conditions and increased transcript level under Al stress in soybean roots (Wu et al., 2018b; Liu et al., 2021). These individual studies strongly suggested that *GmBBE-like43* might participate in soybean root coordinate responses to Al stresses and Pi starvation. However, its functions in root adaptation to these multiple stresses remain unclear. In the present study, genome-wide analyses were first conducted to get an overview of GmBBE-likes in the soybean genome. Subsequently, the expression patterns of *GmBBE-like43* in response to Al stress and P deficiency were determined using qRT-PCR and the effects of alternative *GmBBE-like43* expression on Al tolerance and low-P adaptation were further evaluated in transgenic Arabidopsis and soybean hairy roots. In addition, the OGs-oxidase activity of *GmBBE-like43* was determined *in vivo* using *GmBBE-like43*-overexpressing soybean hairy roots. Our findings will contribute to future research on soybean regarding its coordinated adaptation to Al toxicity and P deficiency on acid soils.

## Materials and Methods

### Plant Material and Growth Conditions

Seeds of soybean (*Glycine max*) genotype YC03-3 were germinated in paper rolls soaked with 1/4 Hogland nutrient solution after being surface-sterilized with 10% (v/v) NaClO solution as described by Liang et al. (2010). Four-day-old seedlings were transplanted to a modified nutrient solution with either 5 or 250 μM KH_2_PO_4._ The other nutrient elements were composed of (in mM): 1.5 KNO_3_, 1.2 Ca (NO_3_)_2_, 0.4 NHNO_3_, 0.025 MgCl_2_, 0.5 MgSO_4_, 0.3 K_2_SO_4_, 0.3 (HN_4_)_2_SO_4_, 0.0015 MnSO_4_, 0.0015 ZnSO_4_, 0.0005 CuSO_4_, 0.00016 (NH_4_)_6_Mo_7_O_24_, 0.0025 NaB_4_O_7_, and 0.04 Fe-Na-EDTA. The nutrient solution was replaced weekly and aerated every 30 min. Plants were harvested to determine the dry weight and total root length at 3, 6, 9, and 12 d after P treatments as described by Wu et al. (2018b) and Zhu et al. (2020). Roots were also harvested for gene expression assays.

To determine the effect of Al stress on *GmBBE-like43* expression, soybean seeds were surface-sterilized and germinated as described above. Four days after germination, the initial root lengths of uniform seedlings were measured. Subsequently, seedlings were subjected to 0.5 mM CaCl_2_ solution (pH 4.2) containing either 0 or 50 μM AlCl_3_ for 0, 12, 24, 48, 72, and 96 h. Root length was measured to calculate the root elongation at the indicated times. Moreover, root tips (0–2 cm) were separately harvested for gene expression assays. For tissue expression analysis of the genes, soybean roots were divided into three segments (0–2 cm, 2–4 cm, and >4 cm) after 24 h of Al treatment. All experiments had at least three biological replicates with five plants for each replicate.

To study the effect of OGs on genes expression in soybean roots under Al toxicity, 4-day-old seedlings were measured for their initial root length and subsequently treated with –Al–OGs (0.5 mM CaCl_2_), –Al+OGs (0.5 mM CaCl_2_, 100 μg mL^−1^ OGs), +Al–OGs (50 μM AlCl_3_), and +Al+OGs (50 μM AlCl_3_, 100 μg mL^−1^ OGs) for 24 h. After treatments, the root length was measured again to calculate the root elongation. The root tips (0–4 cm) were separately harvested to determine gene expression.

### Identification and Bioinformatic Analysis of Soybean GmBBE-Like Genes

The soybean *GmBBE-like* genes were obtained by blast-search analysis in the Phytozomev13 database (https://phytozome-next.jgi.doe.gov/blast-search) with AtBBE-like amino acid sequences as query (Daniel et al., 2015). The physical and chemical properties of GmBBE-like members were analyzed using the ExPASy software (http://www.expasy.org). GmBBE-like protein sequences were aligned with ClustalX and a phylogenetic tree was constructed by MEGA-X using the neighbor-joining method with 1,000 bootstrap replicates. The Gene Structure Display Server2.0 (GSDS) was used for gene structure analysis (Huang et al., 2021). The online CDD (https://www.ncbi.nlm.nih.gov/Structure/cdd/wrpsb.cgi) program and MEME program (https://meme-suite.org/meme/tools/meme) were used to analyze the GmBBE-like conserved domains and motifs, respectively. The conserved domains and motifs were visualized by TBtools (Chen et al., 2020).

### RNA Extraction and qRT- PCR Analysis

Total RNA was extracted using an RNA-solve reagent (OMEGABio-Tek, Norcross, GA, USA) as described previously in Zhu et al. (2020) and treated with RNase-free DNase I (Invitrogen, United States) to eliminate genomic DNA contamination. The amount and purity of RNA in each sample were evaluated by the Nano-drop spectrophotometer (Thermo, United States). Approximately 1 μg of RNA was used to synthesize the first-strand cDNA using MMLV-reverse transcriptase (Promega, USA) following the instructions. The qRT-PCR was performed and analyzed using SYBR Green PCR master mix (Promega, USA) on an ABI7500 real-time PCR system (Thermo Fisher Scientific, Waltham, MA, USA). For target gene expression analysis, the specific primers used are listed in Supplementary Table S1. Soybean housekeeping gene *GmEF1-*α (*Glyma.17G186600*) and Arabidopsis housekeeping gene *AtEF1-*α (*At5G60390*) were used as an endogenous control to normalize the expression of corresponding genes in soybean and Arabidopsis (Zhu et al., 2020; Lin et al., 2021). The relative expression level was calculated by the ratio of the expression level of the target genes to that of the housekeeping gene (Qin et al., 2012).

### GUS Histochemical Analysis and Subcellular Localization Assay of *GmBBE-Like43*

To determine the subcellular localization of *GmBBE-like43*, the coding region of *GmBBE-like43* was amplified with specific primers *GmBBE-like43-GFP-F/R* (Supplementary Table S1), and then cloned into the *pEGAD* vector to produce a *35S::GmBBE-like43-GFP* construct. The *35S::GFP* construct was used as the control. The constructs were subsequently transformed into tobacco epidermal cells (Liu et al., 2016a,b; Zhu et al., 2020). The fluorescent signals were observed *via* a confocal scanning microscope (Zeiss LSM780, Oberkochen, Germany) at 488 nm for GFP (Zhu et al., 2020). Fluorescent images were further processed using Zen2011 software (Carl Zeiss Microscopy, Germany). To further verify the subcellular localization of GmBBE-like43, the *35S::GFP* and *35S::GmBBE-like43-GFP* were also transformed into common bean (*Phaseolus vulgaris*) hairy roots (Liang et al., 2010; Wu et al., 2018b). Propidium iodide (PI) was used to identify the cell wall. Green fluorescence derived from GFP and red fluorescence derived from PI were observed by confocal scanning microscopy at 488 nm and 636 nm (Zeiss LSM780, Oberkochen, Germany), respectively.

The up-stream 2000bp of the *GmBBE-like43* promoter region was amplified with specific primers *GmBBE-like43-GUS-F/R* (Supplementary Table S1). The PCR products were cloned into *pTF102* to produce *pGmBBE-like43::GUS* constructs. The plasmid was subsequently transformed into *Agrobacterium tumefaciens* strain GV3101 and used for Arabidopsis transformation by the floral dip method (Clough and Bent, 1998). The homozygote transgenic Arabidopsis seedlings were either treated with 6.25 μM (–P) and 1.25 mM (+P) KH_2_PO_4_ in 1/2 modified solid MS medium for 6 d or treated with 0 and 5 μM AlCl_3_ in 1/5 Hoagland nutrient solution (pH 4.5, without KH_2_PO_4_, with 0.5 mM CaCl_2_) for 24 h, respectively. After treatments, Arabidopsis seedlings were separately harvested and stained with GUS staining solution (Zhu et al., 2020). After GUS staining, seedlings were pictured using a light microscope (Leica, Germany).

### DAB Staining for Root Surface H_2_O_2_

Uniform soybean hairy roots were subjected to MS nutrient solutions containing either 0 mM or 100 μg mL^−1^ (approximately 54 mM) OGs for 12 h. After treatment, roots were stained with 0.1 mg mL^−1^ DAB in 50 mM Tris buffer, pH 5.0 in a light homoeothermic incubator for 8 h (He et al., 2012). Subsequently, roots were fixed in a solution of 3:1:1 ethanol: lactic acid: glycerol and photographed by a light microscope (Leica, Germany).

### Functional Analysis of *GmBBE-Like43* in Soybean Hairy Roots

The coding region of *GmBBE-like43* was amplified using primers (*GmBBE-like43-OX-F/R*, Supplementary Table S1) and inserted into the modified *pTF101s* vector to produce a *35S::GmBBE-like43* construct. In addition, a 364 bp specific fragment from the *GmBBE-like43* coding region was amplified with specific primers (*GmBBE-like43-RNAi-Asc* I*-F/R* and *GmBBE-like43-RNAi-Bam*H I*-F/R*) (Supplementary Table S1), and PCR products were inserted into the *pFGC5941* vector after digestion by *Asc* I and *Bam*H I, producing *GmBBE-like43-RNAi* construct. Subsequently, the *GmBBE-like43-OX, GmBBE-like43-RNAi*, and their corresponding empty vectors were separately transformed into *Agrobacterium rhizogenes* strain K599, which was further used to infect soybean cotyledons to obtain transgenic hairy roots (Liang et al., 2010; Wu et al., 2018b). Transgenic hairy roots verified by qRT-PCR assays with specific primers *GmBBE-like43-RT-1-F/R* and *GmBBE-like43-RT-2-F/R* (Supplementary Table S1) were used for further analyses.

To investigate the effects of alternative *GmBBE-like43* expression on soybean hairy root growth in response to P treatment, transgenic soybean hairy roots were transplanted to a modified solid MS medium with 0.8% agar (Sigma-Aldrich, USA, CAS: 9002-18-0) containing either 1.25 mM KH_2_PO_4_ (+P) or 10 μM KH_2_PO_4_ (–P) for 14–21 d. The dry weight and total root length of the soybean hairy roots were measured after P treatment. To evaluate the effects of alternated *GmBBE-like43* expression on soybean hairy root growth in response to Al stress, uniform hairy roots were selected, and initial root length was analyzed. Subsequently, hairy roots were subjected to 0 and 100 μM AlCl_3_ in 1/4 modified liquid MS medium (pH 4.2, without KH_2_PO_4_) in a growth chamber with 100 rpm, 28°C for 24 h. After Al treatment, root length was measured. Root elongation (24 h root length−0 h root length) and the relative root elongation (root elongation in 100 μM AlCl_3_/root elongation in 0 μM AlCl_3_ × 100) were evaluated. Each treatment had twelve independent transgenic hairy roots.

### Functional Analysis of *GmBBE-Like43* in Transgenic Arabidopsis

The transgenic Arabidopsis plants with *GmBBE-like43* overexpression were produced as mentioned above. Two homozygous T3 lines (OX1 and OX2) were selected using a qRT-PCR assay with specific primers *GmBBE-like43-RT-3-F/R* (Supplementary Table S1). Columbia-0 wild-type (WT) and *GmBBE-like43*-overexpression seeds were surface-sterilized and then germinated on petri dishes containing modified MS solid medium at 23°C for 16 h /8 h (light /dark). After 4 days, uniform seedlings were selected and treated either with Al stress or P deficiency. For the P treatment, seedlings were transplanted to 1/2 modified solid MS medium with 6.25 μM KH_2_PO_4_ (–P) or 1.25 mM KH_2_PO_4_ (+P). After 9 d, seedlings were harvested to measure root fresh weight and primary root length. For Al treatment, uniform seedlings were transplanted to 1/5 Hoagland nutrient solution (pH 4.5, without KH_2_PO_4_, with 0.5 mM CaCl_2_) with or without 5 μM AlCl_3_ for 48 h. The root length before and after Al treatment was measured to calculate the root elongation and relative root elongation as described above.

### Statistical Analyses

Data analyses and standard error calculations were statistically performed using Microsoft Excel 2010 (Microsoft Company, United States), and boxplots were carried out using Origin 2021b. The student's *t*-test and Duncan's multiple range test were performed with the SPSS program (v21.0; SPSS Institute, United States).

## Results

### Identification and Characterization of *GmBBE-Like* Members

**Figure 1:**
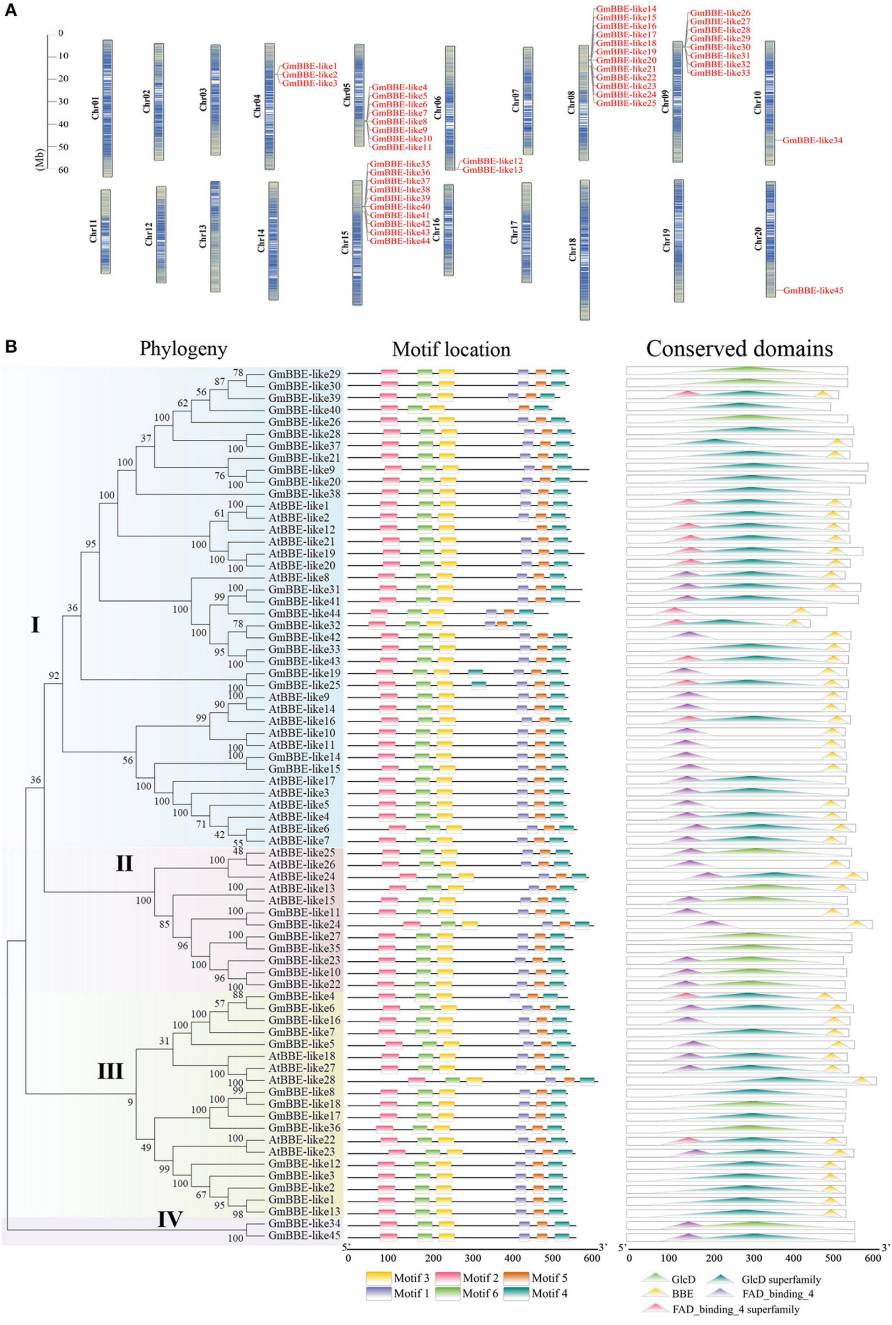
Genes chromosome distribution, phylogeny, and conserved motifs/domains of GmBBE-likes. **(A)** The location of *GmBBE-like* genes in chromosomes. The size of a chromosome is indicated by its relative length and the figure was produced using the TBtools software; **(B)** The phylogenetic analysis and conserved motifs/domains of GmBBE-likes. The phylogenetic tree was created using the MEGA-X program with neighbor-joining method (1000 bootstrap replicates) based on BBE-like protein sequences from soybean and Arabidopsis. The bootstrap values are indicated for major branches as percentages. Conserved motifs and conserved domains in the BBE-like proteins as revealed by MEME analysis.

A survey of the soybean genome (*Glycine max* Wm82. a2. v1) revealed that a total of 45 potential *GmBBE-like* gene sequences had been identified and named *GmBBE-like1* to *GmBBE-like45*, according to their position in the chromosome (Figure 1A). General information on the *GmBBE-like* members was summarized showing that the amino acid residue numbers of GmBBE-like proteins ranged from 443 to 593, their isoelectric point ranged from 5.54 to 9.68, and molecular weight varied from 501 kDa to 665 kDa (Supplementary Table S2). An *in-silico* analysis placed the identified *GmBBE-like* genes on eight different chromosomes. These *GmBBE-like* genes were not randomly distributed in the eight genomes, but most of them were found in tandem arrays of highly related genes (Figure 1A). Conserved domain analysis showed that 36% of the soybean GmBBE-like members had both a FAD-binding domain and a BBE domain. Among them, GmBBE-like43 was grouped in the same clade and showed high similarity with Arabidopsis AtBBE-like members (AtBBE-like1/20/21) that have been reported to contain OGOX activities (Figure 1B).

### Expression of *GmBBE-Like43* Is Regulated by Al Toxicity and Pi Starvation

Our previous proteomic study found that the GmBBE-like43 protein accumulation was increased by P deficiency in the cell wall of soybean roots (Wu et al., 2018b). Moreover, a recent transcriptome study reported that Al stress enhanced the expression level of *GmBBE-like43* (Liu et al., 2021). These studies indicated that *GmBBE-like43* might be involved in the coordinated responses of soybean roots to Al toxicity and P deficiency. Therefore, *GmBBE-like43* was selected for further functional characterization. The qRT-PCR assays were first conducted to analyze *GmBBE-like43* expression patterns in response to Al toxicity and P deficiency.

**Figure 2:**
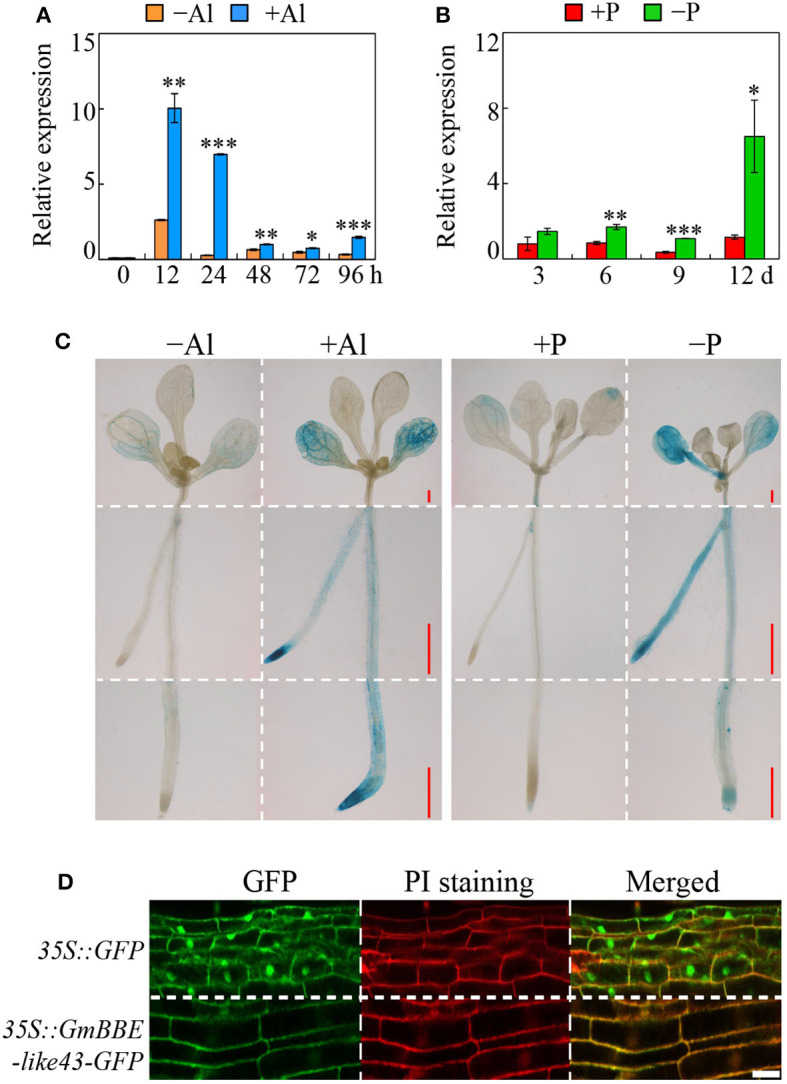
Expression analysis of *GmBBE-like43* and its subcellular localization. **(A)** Relative expression of *GmBBE-like43* in 0-2 cm soybean root tips in response to Al for different time periods; **(B)** Relative expression of *GmBBE-like43* in response to Pi starvation at the indicated times; **(C)** Transgenic Arabidopsis containing the *pGmBBE-like43*::*GUS* and *35S::GUS* were subjected to either Al treatment (0 μM or 5 μM AlCl_3_) for 24 h or P treatment (1.25 mM and 6.25 μM KH_2_PO_4_) for 6 d. Bars = 0.5 mm; **(D)** Subcellular localization analysis of GmBBE-like43 in common bean hairy roots. Red fluorescences were derived from PI indicating the cell wall. Bars = 20 μm. Asterisks indicate a significant difference between either +Al and –Al treatments or –P and +P treatments according to Student's *t*-test: ^*^: *P* < 0.05; ^**^: 0.001 < *P* < 0.01; ^***^: *P* < 0.001.

Results showed that under Al stress, the growth in the primary roots was significantly inhibited after 12 h of Al treatment and maintained a low growth rate (Supplementary Figures 1A,B). Compared to the –Al treatment, the level of *GmBBE-like43* transcript abundance was increased by more than 45.4-fold in the root tips (first 0–2 cm of the root) and 17.7-fold in the middle of the roots (2–4 cm of the root) after 24 h of Al^3+^ exposure, while no significant changes of *GmBBE-like43* expression were observed in the other root regions (>4 cm of the root) (Supplementary Figure 1C). Moreover, the Al-regulated expression of *GmBBE-like43* in root tips was time-course dependent. Without Al stress, the expression level of *GmBBE-like43* remained low during a period of 96 h (Figure 2A). However, after 12 and 24 h of Al treatment, a transient up-regulation of *GmBBE-like43* transcription was observed. The *GmBBE-like43* expression responsive to Al stress was soon decreased after 48 h of Al treatment, remaining low over the next 48 h (Figure 2A). These results indicated that Al^3+^ transiently induced the expression of *GmBBE-like43* in soybean root apexes.

To determine the *GmBBE-like43* expression in response to Pi starvation, soybean seedlings were subjected to different P levels for 3, 6, 9, and 12 days (Supplementary Figure 1D). While the fresh weight of seedlings decreased by 24% after 12 days of low P treatment (Supplementary Figure 1E), the total root length increased by 28 and 33% in 9 and 12 days after low P treatment, respectively (Supplementary Figure 1F). Results of qRT-PCR showed that *GmBBE-like43* expression was initially up-regulated after 6 days of P deficiency. Compared to the P-sufficient roots, the expression of *GmBBE-like43* was 2, 3.1, and 5.6 folds higher in the P-deficient roots after 6, 9, and 12 days of P treatment, respectively (Figure 2B).

### Histochemical Analysis of *GmBBE-Like43* Promoter and Subcellular Localization of GmBBE-Like43

The expression patterns of *GmBBE-like43* in response to Al stress and Pi starvation were further confirmed by the expression of *pGmBBE-like43*::*GUS* in transgenic Arabidopsis plants (Figure 2C). Consistent with the qRT-PCR results, both Al toxicity and Pi starvation strongly enhanced the GUS-staining of transgenic Arabidopsis expressing *pGmBBE-like43*::*GUS* (Figure 2C). Moreover, the subcellular localization of GmBBE-like43 was determined by either expressing *35S::GmBBE-like43-GFP* in common bean hairy roots or transiently expressing it in tobacco leaf epidermis cells. Results showed that GFP fluorescence derived from *35S::GFP* empty vector control was observed all over the cells from both common bean hairy roots and tobacco leaves, whereas, GFP fluorescence derived from 3*5S::GmBBE-like43-GFP* was localized exclusively at the periphery of the cells (Figure 2D, Supplementary Figure 2). Moreover, in the transgenic common bean hairy roots, the 35S::GmBBE-like43-GFP green fluorescence merged well with the red fluorescence derived from PI staining in the cell walls (Figure 2D). It thus confirmed that GmBBE-like43 is a cell wall localized protein.

### Alternative Expression of *GmBBE-Like43* Affects Soybean Hairy Roots Growth in Response to Al Toxicity and Pi Starvation

To characterize the function of *GmBBE-like43* in response to Al toxicity and Pi starvation, transgenic soybean hairy roots with *GmBBE-like43* over-expressed (*GmBBE-like43-*OX) and suppressed (*GmBBE-like43-*RNAi) were generated (Supplementary Figure 3). And transgenic lines expressing empty vector (either *pTF101s* or *pFGC5941*) were used as the corresponding controls (CK-OX and CK-RNAi).

**Figure 3:**
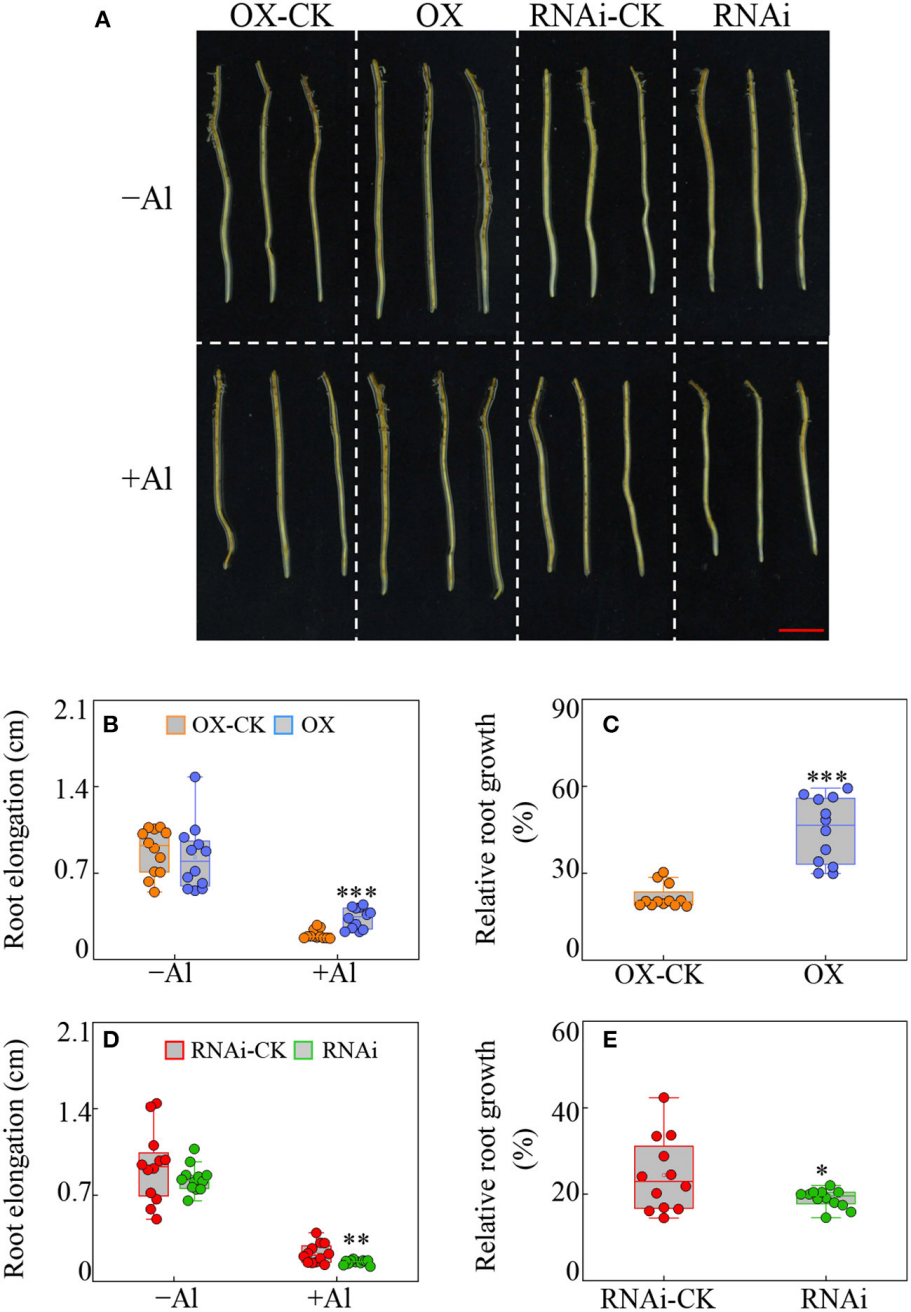
Effects of *GmBBE-like43* overexpression (OX) and RNA-interference (RNAi) on transgenic soybean hairy roots in response to Al treatment. **(A)** Phenotypes of transgenic soybean hairy roots in response to Al stress. **(B–E)** Root elongation **(B,D)** and relative root elongation **(C,E)** of soybean hairy roots with either *GmBBE-like43* overexpressing **(B,C)** or *GmBBE-like43* RNA-interference **(D,E)**. For Al treatment, the uniform soybean hairy roots were transferred to 1/4 modified liquid MS medium (pH 4.2, without KH_2_PO_4_) with either 0 μM (–Al) or 100 μM (+Al) AlCl_3_ for 24 h. Asterisks indicate significant difference between transgenic hairy roots with either *GmBBE-like43* overexpressing or RNA-interference and their corresponding empty vector controls according to Student's *t*-test: ^*^: *P* < 0.05; ^**^: 0.001 < *P* < 0.01; ^***^: *P* < 0.001.

All the transgenic hairy roots were subjected to Al treatment for 24 h and the root elongation and relative root growth were determined. It showed that compared to the corresponding empty vector controls, the root elongation was significantly higher in *GmBBE-like43-*OX lines, but lower in *GmBBE-like43*-RNAi lines in Al treatment, respectively (Figures 3A,B,D). Accordingly, the relative root growth of the *GmBBE-like43-*OX lines and the *GmBBE-like43*-RNAi lines was 1.8-fold higher and 22% lower than that of the corresponding empty vector controls (Figures 3C,E).

**Figure 4:**
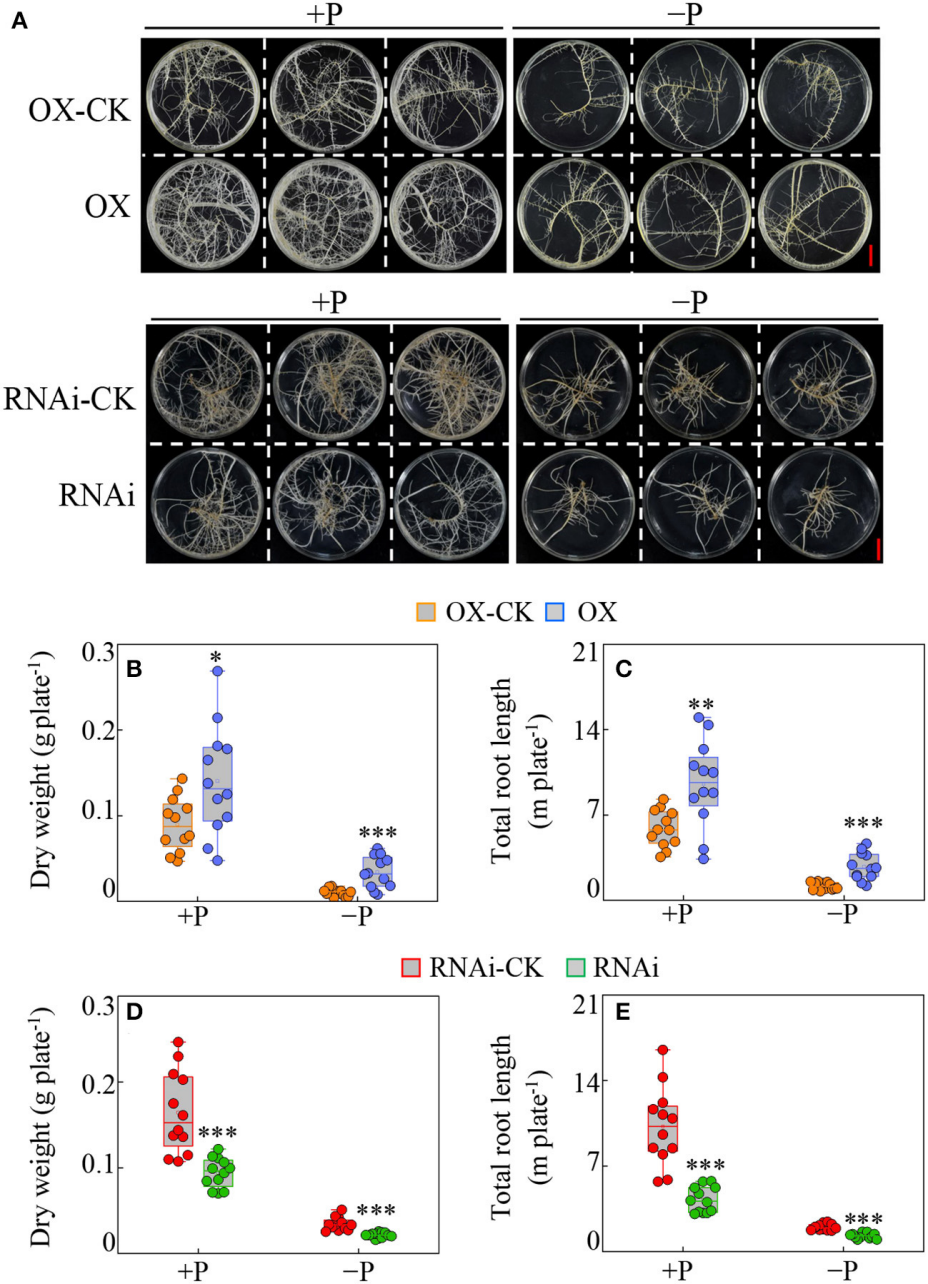
Effects of *GmBBE-like43* overexpression (OX) and RNA-interference (RNAi) on transgenic soybean hairy roots in response to low P treatment. **(A)** Phenotypes of transgenic soybean hairy roots in response to P availability. **(B–E)** Dry weight **(B,D)** and total root length **(C,E)** of soybean hairy roots with either *GmBBE-like43* overexpressing **(B,C)** or *GmBBE-like43* RNA-interference **(D,E)**. For P treatments, uniform soybean hairy roots were selected and transferred to MS medium with either 10 μM (–P) or 1250 μM (+P) KH_2_PO_4_. The hairy roots expressing 35S::*GmBBE-like43* (OX) and *pTF101s* empty vector control (OX-CK) were harvested 14 days after P treatment, while the *GmBBE-like43* RNA-interference (RNAi) and its *pFGC5941* empty vector control (RNAi-CK) were sampled after 21 days of P treatment. Asterisks indicate significant difference between transgenic hairy roots with either *GmBBE-like43* overexpressing or RNA-interference and their corresponding empty vector controls according to Student's *t*-test: ^*^: *P* < 0.05; ^**^: 0.001 < *P* < 0.01; ^***^: *P* < 0.001.

Furthermore, the effects of overexpressed and suppressed *GmBBE-like43* on soybean hairy root growth were detected under different P treatments. Results showed that overexpression of *GmBBE-like43* stimulated transgenic hairy roots growth independent of P availability (Figure 4A). The dry weight of *GmBBE-like43-*OX lines was 1.6 and 3.4 folds higher than the CK-OX control under P sufficient (+P) and P deficient (–P) conditions (Figure 4B). This was confirmed by similar changes in hairy root length in +P treatment (1.6 Folds higher) and –P treatment (2.3 Folds higher) (Figure 4C). In contrast, the hairy root growth was inhibited in *GmBBE-like43-*RNAi lines, as indicated by 43% (+P) and 38% (–P) decrease in root dry weight and 59% (+P) and 35% (–P) decrease in root length (Figures 4D,E).

### Overexpressing *GmBBE-Like43* Enhances Arabidopsis Root Growth in Response to Al Stress and Pi Starvation

To confirm the function of *GmBBE-like43* in root response to Al toxicity and P deficiency, the *35S*::*GmBBE-like43* construct was heterologously expressed in Arabidopsis. The expression of *GmBBE-like43* in two selected transgenic lines (OX1 and OX2) was verified by qRT-PCR (Supplementary Figure 4).

**Figure 5:**
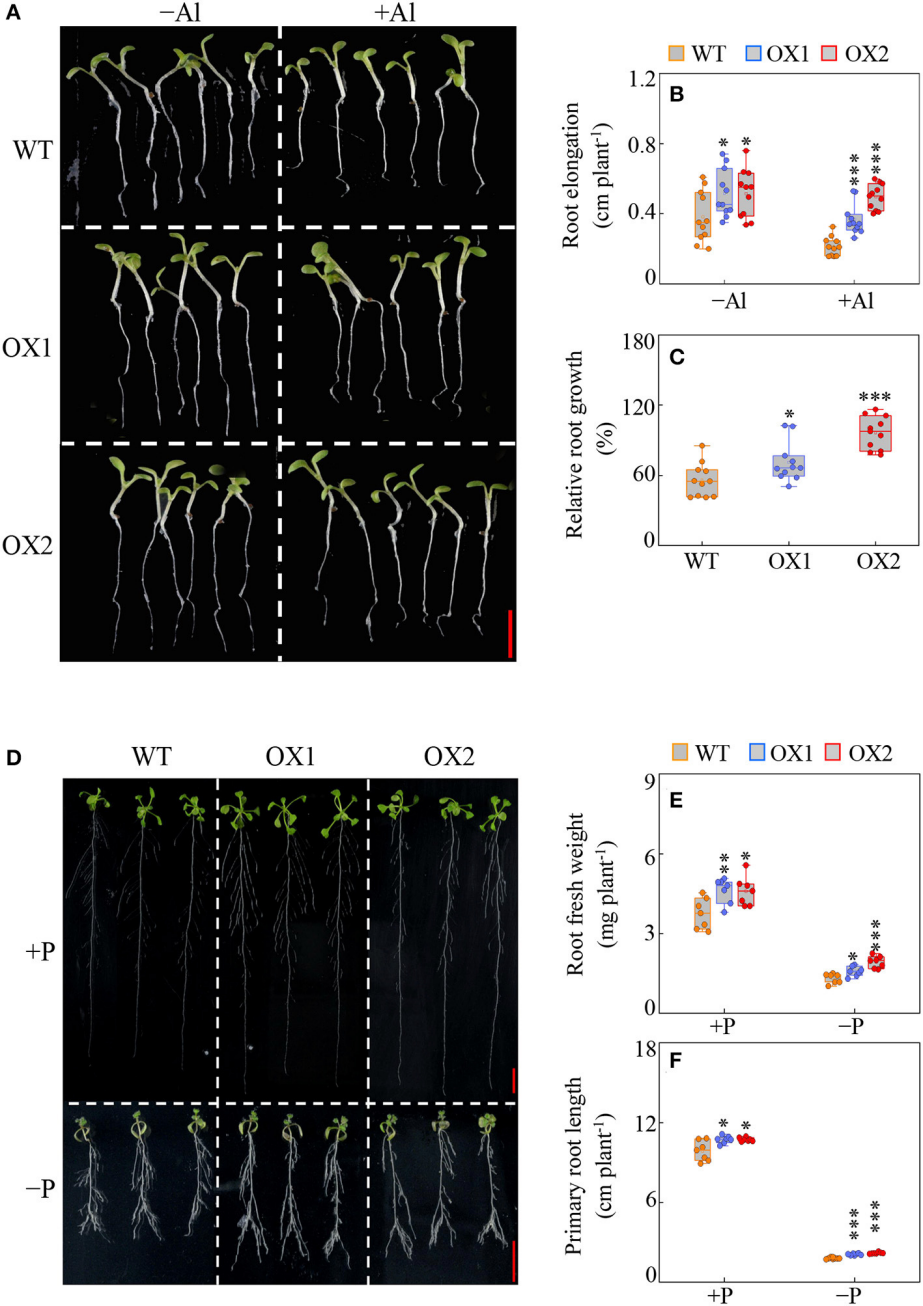
Effects of *GmBBE-like43* overexpression on Arabidopsis root growth in response to Al toxicity and Pi starvation. **(A–C)** Phenotypes **(A)**, root elongation **(B)**, and relative root growth **(C)** of wild-type (WT) and *GmBBE-like43*-overexpression (OX1 and OX2) lines treated with or without 5 μM AlCl_3_ for 48 h, Bar = 0.5 mm; **(D–F)** Phenotypes **(D)**, root fresh weight **(E)**, and primary root length **(F)** of WT, OX1, and OX2 treated with either 1250 μM (+P) or 6.25 μM (–P) KH_2_PO_4_ for 9 days, Bar = 1 cm. Asterisks indicates significant difference between WT and OX according to the Student's *t*-test: ^*^: *P* < 0.05; ^**^: 0.001 < *P* < 0.01; ^***^: *P* < 0.001.

Results showed that in the absence and the presence of Al stress, the root elongation of OX1 and OX2 was higher than the WT control (Figures 5A,B). Furthermore, OX1 and OX2 showed enhanced Al tolerance, as indicated by 27% and 72% increases in the relative root growth compared to WT (Figure 5C).

In addition, similar to the responses of the soybean transgenic hairy roots to P, the transgenic Arabidopsis showed enhanced root growth independent of P availability (Figure 5D). Compared to WT, the root fresh weight of OX1 and OX2 were 23% and 22% higher in +P treatment, and 20% and 47% higher in –P treatment (Figure 5E). The primary root length of *35S*::*GmBBE-like43* transgenic lines was also higher than WT. Under +P condition, the primary root length of both *35S*::*GmBBE-like43* transgenic lines was about 9% higher than WT, while under –P condition, the primary root length of OX1 and OX2 was 17 and 23% higher than WT (Figure 5F).

### Overexpressing *GmBBE-Like43* Attenuated the Effects of Al on Auxin-Responsive Genes Expression

**Figure 6:**
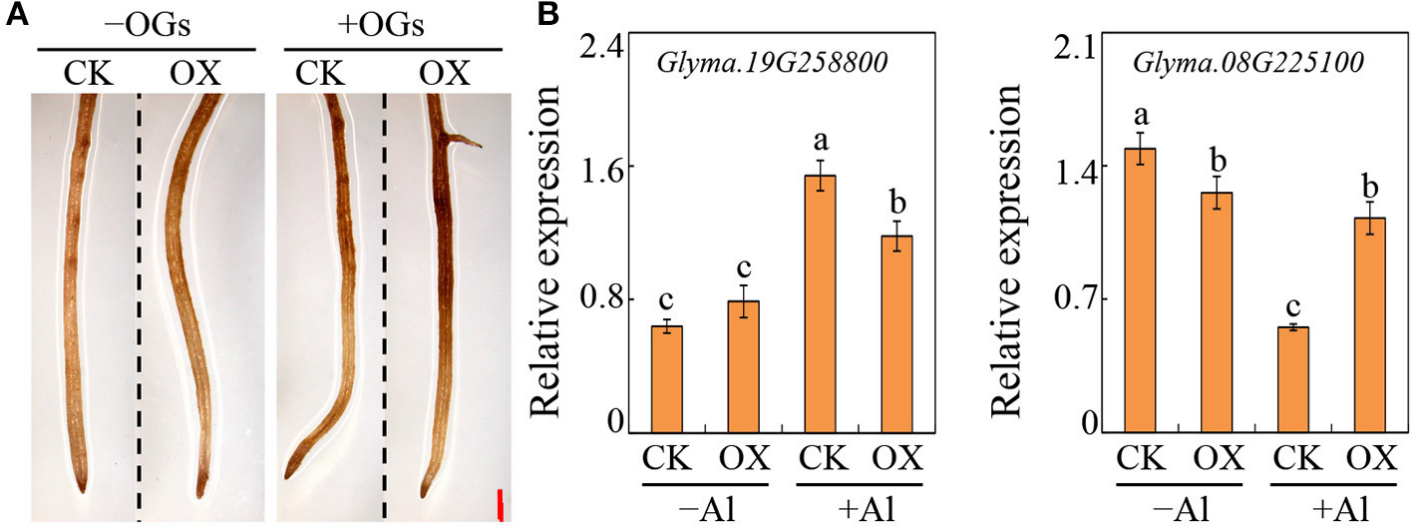
Effects of *GmBBE-like43* overexpression on root surface H_2_O_2_ generation and the expression of two Al-regulated auxin-response genes. **(A)** DAB staining of transgenic soybean hairy roots expressing either *35S::GmBBE-like43* (OX) or empty vector (CK) in treatments with or without OGs; **(B)** Expression of auxin-responsive gene *Glyma.19G258800* and *Glyma.08G225100* in transgenic soybean hairy roots in response to Al treatment. Values are means ± SE of four replicates. Different letters on the bar indicate differences among treatments (*P* < 0.05).

To determine the function of *GmBBE-like43* in OGs oxidation and further alternate the expression of genes involved in root growth regulation, soybean transgenic hairy roots with overexpressed *GmBBE-like43* were generated. Then DAB staining was conducted to determine H_2_O_2_ generation in the transgenic hairy roots. It showed that overexpressing *GmBBE-like43* obviously enhanced the H_2_O_2_ accumulation, both under with and without OGs treatment (Figure 6A). It thus suggested that GmBBE-like43 contained the ability to oxidize OGs.

The expression of auxin-response genes in response to OGs and *GmBBE-like43-*overexpression was further investigated. Based on recent transcriptomic data (Zhao et al., 2020), two Al-regulated *SAUR-like* genes (*Glyma.08G225100* and *Glyma.19G258800*) were selected. Their expressions were first determined by qRT-PCR assays in soybean primary roots treated with combinations of Al and OGs. Consistent with the transcriptome results, the expression of *Glyma.19G258800* was enhanced and *Glyma.08G225100* was suppressed by Al stress in soybean roots. Moreover, when treated with +Al+OGs, the expression of *Glyma.19G258800* was even higher and *Glyma.08G225100* was lower than that in +Al treatment (Supplementary Figure 5). Moreover, in overexpressed *GmBBE-like43* soybean hairy roots, the suppression of *Glyma.08G225100* by Al stress was alleviated, as well as the Al-regulated enhancement of *Glyma.19G258800* expressions (Figure 6B). These results indicated that GmBBE-like43 was able to antagonize the effects of Al on the expression of two *SAUR-like* genes, probably by alleviating OGs activity.

## Discussion

High Al and low P are two major limiting factors for crop production in acid soils, which constitute up to 40% of arable land worldwide (Kochian et al., 2004). Since rhizotoxic Al and P deficiency coexist in acid soils, it is clear that plant roots have to deal with these two stresses simultaneously during their growth in acidic soils (Liao et al., 2006; Liang et al., 2013). Although studies of the molecular mechanisms have been conducted focusing primarily on Al tolerance or occasionally on P efficiency, few studies have simultaneously considered P availability and Al toxicity together (Zheng et al., 2005; Liao et al., 2006; Ligaba et al., 2007; Liang et al., 2013). Cell walls in plant roots are directly connected to the rhizosphere environment. The structural and functional integrity of the cell wall needs to be constantly monitored and finetuned allowing plants to survive under stress (Rui and Dinneny, 2020). Pectin in the cell wall is a group of complex polysaccharides that are targeted by Al (Cosgrove, 2005). Increased esterified homogalacturonans (HGs) and decreased methyl-esterified HGs were observed in many plant species under Al stress (Yang et al., 2008, 2011, 2013; Jaskowiak et al., 2019). Compared to Al stress, fewer studies have been conducted to determine the pectin methylation level in cell walls in response to Pi-starvation. A recent report showed that increased pectin levels with a low degree of methyl-esterification were observed under low P conditions in mature internodes of grapevine (*Vitis vinifera*) (Fernandes et al., 2016). These results suggest that plant cell wall undergoes pectin demethyl-esterification under both Al toxicity and P deficient conditions. This modification further increases the possibility of the production of active de-methylated oligogalacturonides (OGs), which were found to be deleterious with high concentrations (Wolf et al., 2012; Ferrari et al., 2013; Benedetti et al., 2015). Therefore, a mechanism to control OGs homeostasis under Al and P stresses might exist to prevent their prolonged impairment.

The BBE-like proteins belong to the superfamily of FAD-linked oxidases, which have been reported to inactivate the elicitor activity of OGs and maintain their homeostasis in the cell wall (Benedetti et al., 2018). It is found that at least four BBE-like enzymes in Arabidopsis contain an OG-oxidizing (OGOX) activity that is accompanied by H_2_O_2_ production (Daniel et al., 2017; Benedetti et al., 2018). Reviewing previous proteomic and transcriptome studies, we noticed that a BBE-like member (GmBBE-like43) in soybean showed enhanced protein accumulation under Pi starvation conditions and increased transcript level under Al toxicity (Wu et al., 2018b; Liu et al., 2021). In this present study, qRT-PCR assays were used to confirm the expression of *GmBBE-like43* in response to Al and low P stresses. Similar results were obtained when expression of *GmBBE-like43* was transiently enhanced by Al toxicity in soybean root tips and increased in roots after 6 days of Pi starvation (Figures 2A,B and Supplementary Figure 1C). Further histochemical analysis of the GmBBE-like43 promoter also proved this (Figure 2C). Therefore, it is suggested that soybean *BBE-like* genes, such as *GmBBE-like43*, might be involved in soybean roots' coordinated adaptation to both Al and low P stresses.

To test this hypothesis, we first characterized all the *GmBBE-like* genes in the soybean genome and found a total of 45 GmBBE-like members in the soybean genome, most of which are located as tandem arrays in five chromosomes (Figure 1A). Among them, GmBBE-like43 showed high similarity with and the same protein structures as AtBBE-like members (AtBBE-like1/20/21) that exhibited OGOX activities but had low similarity with AtBBE-like8, which was identified as a cellulose oligomer oxidase (Figure 1B) (Benedetti et al., 2018; Locci et al., 2019). It thus indicated that GmBBE-like43 might have the same enzyme functions in OG-oxidation as its homologs in Arabidopsis. Considering the H_2_O_2_ production in the OG-oxidation reaction (Daniel et al., 2017; Benedetti et al., 2018), we generated *GmBBE-like43* overexpressing soybean hairy roots and used DAB staining to determine whether GmBBE-like43 was able to oxidize OGs and produce H_2_O_2_. It showed that *GmBBE-like43* overexpressing enhanced DAB staining on surfaces of the transgenic soybean hairy roots, especially with OGs application (Figure 6). Together with the cell wall localization of GmBBE-like43 (Figure 2D and Supplementary Figure 2) (Wu et al., 2018b), we suggested that *GmBBE-like43* had OGOX activities and was able to oxidate OGs in the cell wall.

As far as the deleterious effects of the active OGs are concerned, early observations showed that external treatment of plant tissues with high amounts of OGs caused tissue necrosis and disturbed plant developmental-related processes (Cervone et al., 1987; Branca et al., 1988; Bellincampi et al., 1993; Altamura et al., 1998). These adverse effects also confirmed that elevated levels of released OGs in transgenic Arabidopsis plants caused the accumulation of salicylic acid, reduced growth, and eventually led to plant death (Benedetti et al., 2015). Using the transgenic Arabidopsis with *OGOX1* (*At4g20830*) overexpression, Benedetti et al. (2018) demonstrated that OGs oxidation occurs enzymatically *in vivo*, and oxidized OGs possess lower eliciting activity than non-oxidized OGs. Therefore, we speculated that if *GmBBE-like43* is expressed at the right time, it might be able to convert the active OGs into inactive forms (e.g., oxidized-OGs) to prevent the deleterious effects of OGs hyper-accumulation induced by either Al toxicity or Pi starvation. This hypothesis was further confirmed when the overexpression of *GmBBE-like43* provided the Al tolerance and adaptation to low P in transgenic soybean hairy roots and transgenic Arabidopsis (Figures 3–5). On the other hand, an interference with the *GmBBE-like43* expression in the transgenic soybean hairy roots reduced both the Al tolerance and low P adaptation (Figures 3, 4).

Indeed, OGs regulate several developmental-related processes including interfering with pea stem elongation (Branca et al., 1988), as well as inhibiting root formation in both tobacco (*Nicotiana taba*cum) and Arabidopsis (Bellincampi et al., 1993; Altamura et al., 1998; Savatin et al., 2011). In these cases, their action is antagonistic to the effects of exogenous auxin. Moreover, the transcript accumulation of several auxin-induced genes, as well as the activation of the synthetic auxin-responsive promoter DR5, are inhibited by OGs (Savatin et al., 2011). Metabolism and polar distribution of auxin have been reported to be involved in root growth inhibition due to Al toxicity through auxin transporters and auxin response factors (Ranjan et al., 2021). If OGs have antagonistic effects on auxin responses, they might also regulate the expression of auxin response genes, which is modulated by the Al treatment. Accordingly, the present study showed that the application of OGs enhanced the Al-induced effects on the expression of *two* soybean *SAUR-like* genes, which were either Al-up-regulated or Al-down-regulated (Supplementary Figure 5) (Zhao et al., 2020). However, in comparison, *GmBBE-like43* had the opposite effect on the Al-regulated expression of *SAUR-like* genes (Figure 6). It thus confirmed that the inactivation of OGs in cell walls mediated by *GmBBE-like43* is one of the soybean's Al-tolerance mechanisms. However, the knowledge about how *GmBBE-like43* functions in soybean's adaptation to Pi-starvation is still limited. Further characterization of *GmBBE-like43* in P-modulated hormone-signaling and cell wall metabolisms may help to elucidate this aspect of abiotic adaptation.

## Data Availability Statement

All datasets generated for this study are included in the article/Supplementary Material.

## Author Contributions

CL and JT conceived and designed the experiments. QC and JL performed the experiments. QC, JL, GL, XL, and KC analyzed the data. CL, JT, QC, and JL wrote the manuscript. All authors have read and approved the final manuscript.

## Funding

This work was supported by a grant from the National Key Research and Development Program of China (2021YFF1000500), Major Program of Guangdong Basic and Applied Research (2019B030302006), the National Natural Science Foundation of China (32172659, 32172658, and 31872164), and the Natural Science Foundation of Guangdong Province of China (2021A1515010826 and 2020A1515110261).

## Conflict of Interest

The authors declare that the research was conducted in the absence of any commercial or financial relationships that could be construed as a potential conflict of interest.

## Publisher's Note

All claims expressed in this article are solely those of the authors and do not necessarily represent those of their affiliated organizations, or those of the publisher, the editors and the reviewers. Any product that may be evaluated in this article, or claim that may be made by its manufacturer, is not guaranteed or endorsed by the publisher.
