# Supplementary material for: A Berberine Bridge Enzyme-Like Protein, GmBBE-like43, Confers Soybean's Coordinated Adaptation to Aluminum Toxicity and Phosphorus Deficiency
Source: Front Plant Sci. 2022 Aug 8;13:947986. doi: 10.3389/fpls.2022.947986 (PMC9393741; doi:10.3389/fpls.2022.947986)
Supplement: Supplementary file 1 [file Data_Sheet_1.zip › data sheet/Supplementary figures-2022.7.22.pdf]

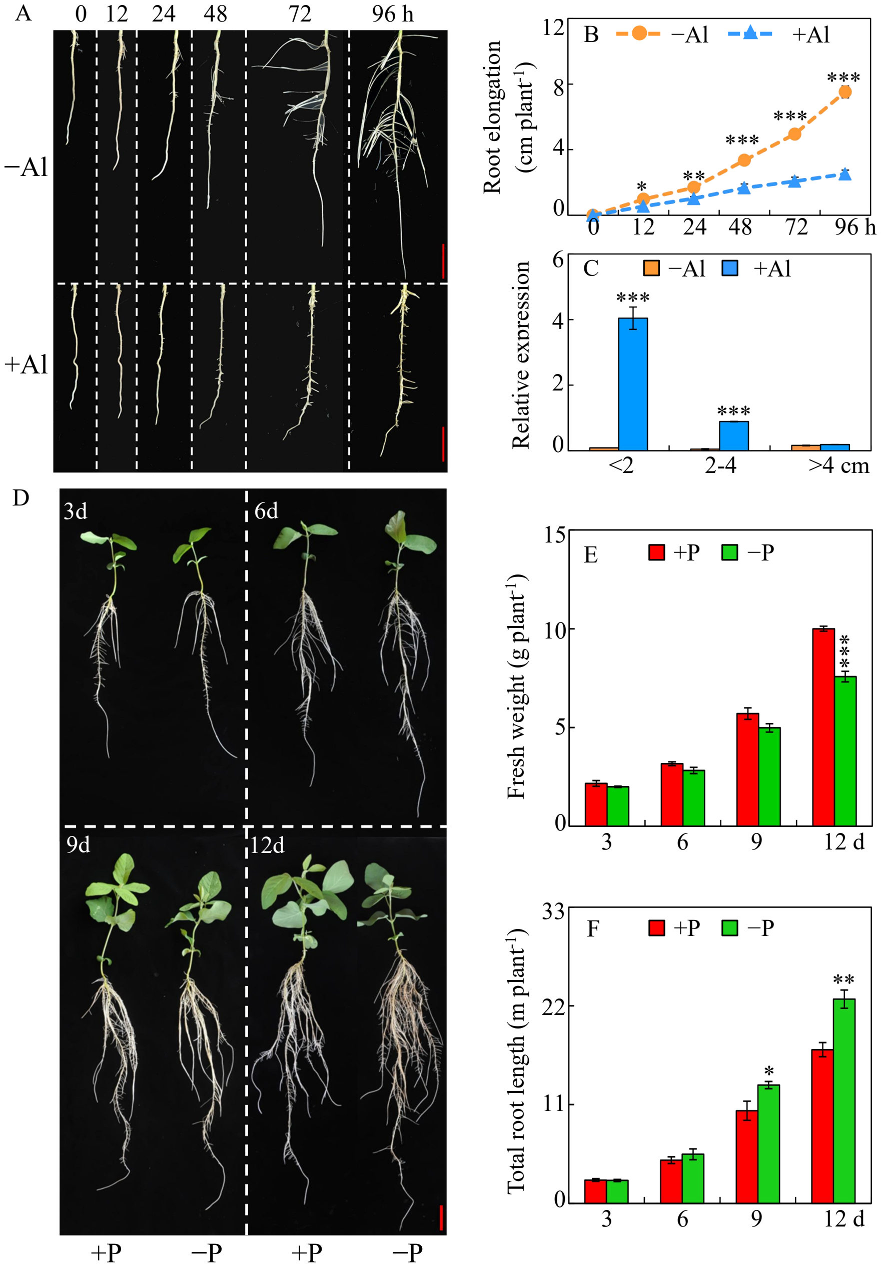

Supplementary Figure 1. Effect of Al stress and low Pi availability on soybean root growth and GmBBE-like43 expression. (A) Phenotype of soybean roots treated with (+Al) or without (−Al) 50 μM $AlCl_3$ for 0, 12, 24, 48, 72 and 96 h. Bar=2 cm; (B) Root elongation; (C) Relative expression of *GmBBE-like43* in different soybean root segments (0-2 cm, 2-4 cm and >4 cm) treated with or without 50 μM $AlCl_3$ for 24 h; (D-F) Phenotype (D), fresh weight (E) and total root length (F) of soybean plants treated with either with 5 μM (−P) or 250 μM (+P) KH_2PO_4 for 3, 6, 9 and 12 d. Bar=5 cm. Each bar represents the mean of at least three independent replicates with standard error. Asterisks indicate significant difference between either +Al and −Al treatments or −P and +P treatments according to Student's *t*-test : *: $P < 0.05$; **: $0.001 < P < 0.01$; ***: $P < 0.001$.

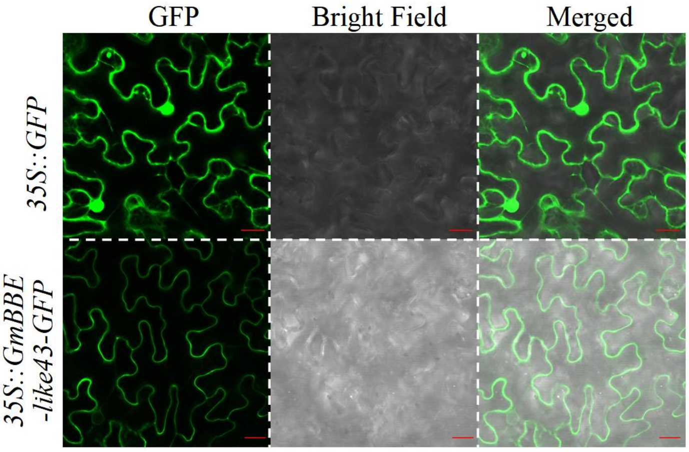

Supplementary Figure 2. Subcellular localization of GmBBE-like43 in tobacco epidermal cells. Scale bars are 20 μm.

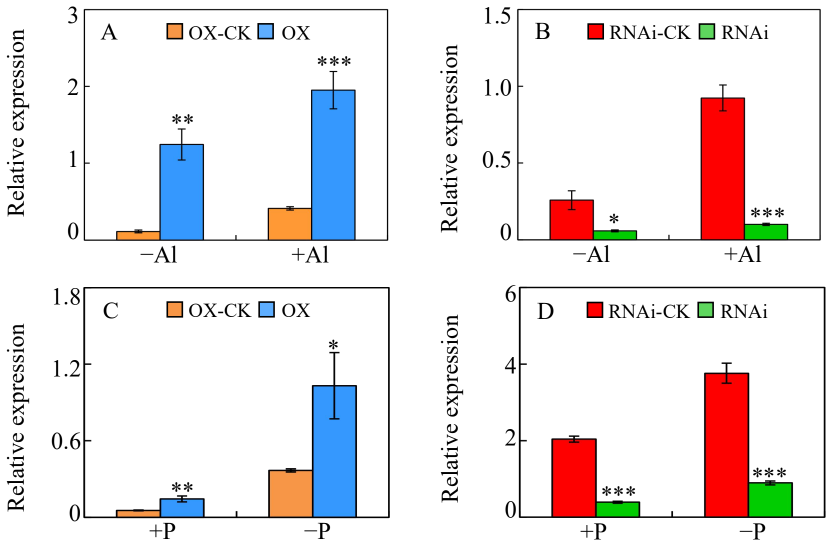

Supplementary Figure 3. qRT-PCR analysis of the *GmBBE-like43* transcripts in transgenic soybean hairy roots in different treatments. (A, B) Relative expression of *GmBBE-like43* in transgenic soybean hairy roots with either *GmBBE-like43* overexpressing (A) or *GmBBE-like43* RNA-interference (B) and their corresponding empty vector controls (OX-CK, RNAi-CK) under Al treatment. (C, D) Relative expression of *GmBBE-like43* in transgenic soybean hairy roots with either *GmBBE-like43* overexpressing (C) or *GmBBE-like43* RNA-interference (D) and their corresponding empty vector controls (OX-CK, RNAi-CK) under P treatment. Data are means of four replicates with standard errors. Asterisks indicate significant difference between transgenic hairy roots with either *GmBBE-like43* overexpressing or RNA-interference and their corresponding empty vector controls according to Student's *t*-test: *: $P < 0.05$; **: $0.001 < P < 0.01$; ***: $P < 0.001$.

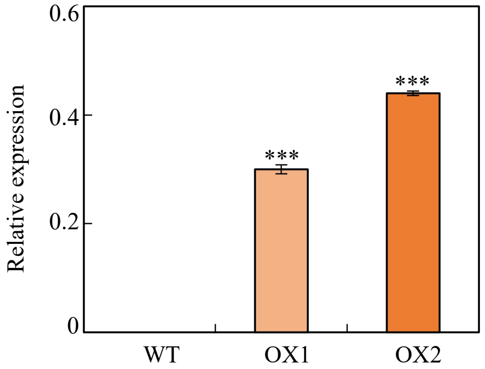

Supplementary Figure 4. Relative expression of *GmBBE-like43* in roots of wild type (WT) and transgenic Arabidopsis lines with *GmBBE-like43* overexpression (OX1 and OX2). Data are means of four replicates with standard errors. Asterisks indicate significant differences between WT and OX by Student's *t*-test: ***: $P < 0.001$.

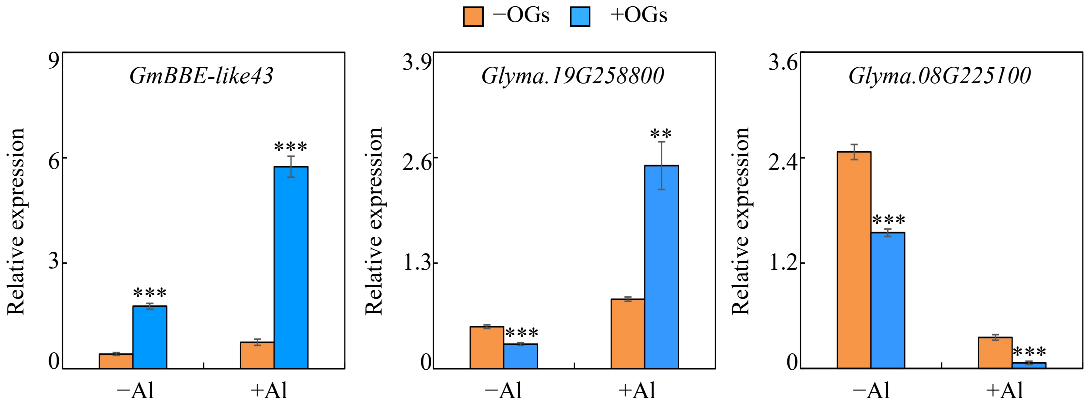

Supplementary Figure 5. Effects of OGs on the transcripts accumulation of *GmBBE-like43* and two Al-regulated auxin-response genes (*Glyma.19G258800* and *Glyma.08G225100*) under Al toxicity. Data are means of four replicates with standard errors. Asterisks indicate significant difference between with and without OGs treatments according to Student's *t*-test: **: $0.001 < P < 0.01$; ***: $P < 0.001$.
