# Supplementary material for: A Berberine Bridge Enzyme-Like Protein, GmBBE-like43, Confers Soybean's Coordinated Adaptation to Aluminum Toxicity and Phosphorus Deficiency
Source: Front Plant Sci. 2022 Aug 8;13:947986. doi: 10.3389/fpls.2022.947986 (PMC9393741; doi:10.3389/fpls.2022.947986)
Supplement: Supplementary file 1 [file Data_Sheet_1.zip › data sheet/Supplementary tables-2022.6.10.pdf]

TABLE S1. Primers used for qRT-PCR and vector construction

Primer name	Forward primer(5’-3’)	Reverse primer(5’-3’)
GmBBE-like43-RT-1	CGTGAACCATTCTGAGCCTTC	AATGGAACCTCTGCCACGTAAG
GmBBE-like43-RT-2	GGACGTTGTGAACGGTACACG	AGGATGCAATGTCTATGTTGTCC
GmBBE-like43-RT-3	CTCCTTTCCCTCATCGAGCTG	TCCATAAACTCTCCCTTCAGCG
GmEF1-α	TGCAAAGGAGGCTGCTAACT	CAGCATCACCGTTCTTCAAA
AtEF1-α	GTCGATTCTGGAAAGTCGACC	AATGTCAATGGTGATACCACGC
GmBBE-like43-GFP	CTCTAGCGCTACCGGTATGGGAGTCCTTTCTTCTCA	CATGGTGGCGACCGGTCGACCCTTCCTATATGACAGCG
GmBBE-like43-GUS	CGGAATTCCACGATGGAGTGCAAAAGCAT	AAGGATCCTTTGGCTTATCCCAATGATGAG
GmBBE-like43-OX	GGGACACTAGTATCGATATGGGAGTCCTTTCTTCTCA	ATCTCGACGGTATCGATACCCTTCCTATATGACAGCG
Gm BBE-like43-RNAi-Asc I	ACAATTACCATGGGGCGCGCCGGTGTGTCTTACGTGGCAGA	AAATCATCGATTGGGCGCGCCCAAAGCTAGCTCCACCACCA
Gm BBE-like43-RNAi-BamH I	ATTTGCAGGTATTTGGATCCCAAAGCTAGCTCCACCACCA	TCTAGACTCACCTAGGATCCGGTGTGTCTTACGTGGCAGA
Glyma.19G258800	TCAAACAAGTTCCGCAGACA	TGCTTCAGGTAATTCACGGG
Glyma.08G225100	GAGGGTGTCCTCAAAATCCC	GACGCATTGAAGCCAAACTC

TABLE S2. General information on *GmBBE-like* members in soybean.

Name	Locus	Length of ORF (bp)	Number of amino acids (aa)	Isoelectric Point	Molecular Weight (kD)
GmBBE-like1	Glyma.04G113300	1587	528	6.55	60
GmBBE-like2	Glyma.04G113400	1587	528	7.28	60
GmBBE-like3	Glyma.04G113500	1587	528	8.31	60
GmBBE-like4	Glyma.05G122000	1593	530	7.68	59
GmBBE-like5	Glyma.05G124800	1650	549	8.59	61
GmBBE-like6	Glyma.05G124900	1644	547	6.16	61
GmBBE-like7	Glyma.05G125000	1611	536	9.25	61
GmBBE-like8	Glyma.05G125200	1593	530	8.75	59
GmBBE-like9	Glyma.05G125600	1749	582	6.48	64
GmBBE-like10	Glyma.05G125800	1596	531	9.09	60
GmBBE-like11	Glyma.05G125900	1605	534	9.63	60
GmBBE-like12	Glyma.06G321500	1584	527	8.91	60
GmBBE-like13	Glyma.06G321600	1590	529	7.68	60
GmBBE-like14	Glyma.08G059000	1593	530	8.87	59
GmBBE-like15	Glyma.08G059100	1596	531	8.38	60
GmBBE-like16	Glyma.08G079900	1620	539	7.64	61
GmBBE-like17	Glyma.08G080000	1584	527	8.43	58
GmBBE-like18	Glyma.08G080100	1590	529	9.13	59
GmBBE-like19	Glyma.08G080200	1596	531	8.36	60
GmBBE-like20	Glyma.08G080400	1734	577	6.85	64
GmBBE-like21	Glyma.08G080500	1620	539	8.57	60
GmBBE-like22	Glyma.08G080600	1584	527	8.81	59
GmBBE-like23	Glyma.08G080700	1572	523	9.57	58
GmBBE-like24	Glyma.08G080900	1782	593	9.67	67
GmBBE-like25	Glyma.08G112600	1608	535	6.25	60
GmBBE-like26	Glyma.09G022600	1602	533	9.68	60
GmBBE-like27	Glyma.09G026900	1632	543	6.58	61
GmBBE-like28	Glyma.09G027000	1647	548	9.59	61
GmBBE-like29	Glyma.09G027200	1602	533	9.22	59
GmBBE-like30	Glyma.09G027300	1602	533	8.75	59
GmBBE-like31	Glyma.09G028100	1698	565	8.68	64
GmBBE-like32	Glyma.09G028200	1332	443	9.45	50
GmBBE-like33	Glyma.09G028300	1614	537	9.18	60
GmBBE-like34	Glyma.10G177100	1653	550	5.73	61
GmBBE-like35	Glyma.15G132600	1632	543	8.04	61
GmBBE-like36	Glyma.15G132700	1569	522	8.48	58
GmBBE-like37	Glyma.15G132800	1635	544	9.02	61
GmBBE-like38	Glyma.15G132900	1614	537	6.52	60
GmBBE-like39	Glyma.15G133100	1536	511	8.61	57
GmBBE-like40	Glyma.15G133200	1479	492	9.68	55
GmBBE-like41	Glyma.15G133900	1680	559	8.61	63
GmBBE-like42	Glyma.15G134200	1626	541	9.56	61
GmBBE-like43	Glyma.15G134300	1608	535	9.17	60
GmBBE-like44	Glyma.15G153700	1452	483	8.22	55
GmBBE-like45	Glyma.20G213100	1653	550	5.54	61
