# Supplementary material for: A Berberine Bridge Enzyme-Like Protein, GmBBE-like43, Confers Soybean's Coordinated Adaptation to Aluminum Toxicity and Phosphorus Deficiency
Source: Front Plant Sci. 2022 Aug 8;13:947986. doi: 10.3389/fpls.2022.947986 (PMC9393741; doi:10.3389/fpls.2022.947986)
Supplement: Supplementary file 1 [file Data_Sheet_1.zip › data sheet/Supplementary figures-2022.7.22.doc]

0 12	24 48	72	96 h

a

−Al

+Al

A

Relative expression

<2	2-4	>4 cm

***

***

6

4

2

0

−Al	+Al

C

Root elongation

(cm plant^-1^)

0	12 24 48	72	96 h

12

8

4

0

−Al	+Al

***

***

**

*

***

B

D

3d

9d

12d

6d

+P	−P	+P	−P

3	6	9	12 d

F

Total root length (m plant^-1^)

33

22

11

0

**

*

+P	−P

15

10

5

0

Fresh weight (g plant^-1^)

***

3	6	9	12 d

+P	−P

E

**Supplementary Figure 1.** Effect of Al stress and low Pi availability on soybean root growth and GmBBE-like43 expression. (A) Phenotype of soybean roots treated with (+Al) or without (−Al) 50 μM AlCl_3_ for 0, 12, 24, 48, 72 and 96 h. Bar=2 cm; (B) Root elongation; (C) Relative expression of *GmBBE-like43* in different soybean root segments (0-2 cm, 2-4 cm and >4 cm) treated with or without 50 μM AlCl_3_ for 24 h; (D-F) Phenotype (D), fresh weight (E) and total root length (F) of soybean plants treated with either with 5 μM (−P) or 250 μM (+P) KH_2_PO_4_ for 3, 6, 9 and 12 d. Bar=5 cm. Each bar represents the mean of at least three independent replicates with standard error. Asterisks indicate significant difference between either +Al and −Al treatments or −P and +P treatments according to Student’s *t*-test : *: *P* < 0.05; **: 0.001 < *P* < 0.01; ***: *P* < 0.001.

GFP	Bright Field	Merged

*35S::GmBBE*

*-like43-GFP*

*35S::GFP*

**Supplementary Figure 2.** Subcellular localization of GmBBE-like43 in tobacco epidermal cells*.* Scale bars are 20 µm.

3

2

1

0

−Al	+Al

***

**

Relative expression

A

OX-CK	OX

Relative expression

1.5

1.0

0.5

0

−Al	+Al

***

*

B

RNAi-CK	RNAi

1.8

1.2

0.6

0

+P	−P

**

*

C

Relative expression

OX-CK	OX

Relative expression

6

4

2

0

+P	−P

***

***

D

RNAi-CK	RNAi

**Supplementary Figure 3.** qRT-PCR analysis of the *GmBBE-like43* transcripts in transgenic soybean hairy roots in different treatments. (A, B) Relative expression of *GmBBE-like43* in transgenic soybean hairy roots with either *GmBBE-like43* overexpressing (A) or *GmBBE-like43* RNA-interference (B) and their corresponding empty vector controls (OX-CK, RNAi-CK) under Al treatment. (C, D) Relative expression of *GmBBE-like43* in transgenic soybean hairy roots with either *GmBBE-like43* overexpressing (C) or *GmBBE-like43* RNA-interference (D) and their corresponding empty vector controls (OX-CK, RNAi-CK) under P treatment. Data are means of four replicates with standard errors. Asterisks indicate significant difference between transgenic hairy roots with either *GmBBE-like43* overexpressing or RNA-interference and their corresponding empty vector controls according to Student’s *t*-test: *: *P* < 0.05; **: 0.001 < *P* < 0.01; ***: *P* < 0.001.

Relative expression

0.6

0.4

0.2

0

WT	OX1	OX2

***

***

**Supplementary Figure 4.** Relative expression of *GmBBE-like43* in roots of wild type (WT) and transgenic Arabidopsis lines with *GmBBE-like43* overexpression (OX1 and OX2). Data are means of four replicates with standard errors. Asterisks indicate significant differences between WT and OX by Student’s *t*-test: ***: *P* < 0.001.

9

6

3

0

Relative expression

−Al	+Al

***

***

3.9

2.6

1.3

0

***

**

Relative expression

−Al	+Al

3.6

2.4

1.2

0

***

***

Relative expression

−Al	+Al

−OGs	+OGs

*GmBBE-like43*

*Glyma.08G225100*

*Glyma.19G258800*

**Supplementary Figure 5.** Effects of OGs on the transcripts accumulation of *GmBBE-like43* and two Al-regulated auxin-response genes (*Glyma.19G258800* and *Glyma.08G225100*) under Al toxicity. Data are means of four replicates with standard errors. Asterisks indicate significant difference between with and without OGs treatments according to Student’s *t*-test: **: 0.001 < *P* < 0.01; ***: *P* < 0.001.
