# Supplementary material for: A Berberine Bridge Enzyme-Like Protein, GmBBE-like43, Confers Soybean's Coordinated Adaptation to Aluminum Toxicity and Phosphorus Deficiency
Source: Front Plant Sci. 2022 Aug 8;13:947986. doi: 10.3389/fpls.2022.947986 (PMC9393741; doi:10.3389/fpls.2022.947986)
Supplement: Supplementary file 1 [file Data_Sheet_1.zip › data sheet/Supplementary tables-2022.6.10.doc]

TABLE S1. Primers used for qRT-PCR and vector construction

| Primer name | Forward primer(5’-3’) | Reverse primer(5’-3’) |
| --- | --- | --- |
| *GmBBE-like43-RT-1* | CGTGAACCATTCTGAGCCTTC | AATGGAACCTCTGCCACGTAAG |
| *GmBBE-like43-RT-2* | GGACGTTGTGAACGGTACACG | AGGATGCAATGTCTATGTTGTCC |
| *GmBBE-like43-RT-3* | CTCCTTTCCCTCATCGAGCTG | TCCATAAACTCTCCCTTCAGCG |
| *GmEF1-α* | TGCAAAGGAGGCTGCTAACT | CAGCATCACCGTTCTTCAAA |
| *AtEF1-α* | GTCGATTCTGGAAAGTCGACC | AATGTCAATGGTGATACCACGC |
| *GmBBE-like43-GFP* | CTCTAGCGCTACCGGTATGGGAGTCCTTTCTTCTCA | CATGGTGGCGACCGGTCGACCCTTCCTATATGACAGCG |
| *GmBBE-like43-GUS* | CGGAATTCCACGATGGAGTGCAAAAGCAT | AAGGATCCTTTGGCTTATCCCAATGATGAG |
| *GmBBE-like43-OX* | GGGACACTAGTATCGATATGGGAGTCCTTTCTTCTCA | ATCTCGACGGTATCGATACCCTTCCTATATGACAGCG |
| *Gm BBE-like43-RNAi-Asc* I | ACAATTACCATGGGGCGCGCCGGTGTGTCTTACGTGGCAGA | AAATCATCGATTGGGCGCGCCCAAAGCTAGCTCCACCACCA |
| *Gm BBE-like43-RNAi-BamH* I | ATTTGCAGGTATTTGGATCCCAAAGCTAGCTCCACCACCA | TCTAGACTCACCTAGGATCCGGTGTGTCTTACGTGGCAGA |
| *Glyma.19G258800* | TCAAACAAGTTCCGCAGACA | TGCTTCAGGTAATTCACGGG |
| *Glyma.08G225100* | GAGGGTGTCCTCAAAATCCC | GACGCATTGAAGCCAAACTC |

TABLE S2. General information on *GmBBE-like* members in soybean.

| Name | Locus | Length of ORF (bp) | Number of amino acids (aa) | Isoelectric Point | Molecular Weight (kD) |
| --- | --- | --- | --- | --- | --- |
| GmBBE-like1 | Glyma.04G113300 | 1587 | 528 | 6.55 | 60 |
| GmBBE-like2 | Glyma.04G113400 | 1587 | 528 | 7.28 | 60 |
| GmBBE-like3 | Glyma.04G113500 | 1587 | 528 | 8.31 | 60 |
| GmBBE-like4 | Glyma.05G122000 | 1593 | 530 | 7.68 | 59 |
| GmBBE-like5 | Glyma.05G124800 | 1650 | 549 | 8.59 | 61 |
| GmBBE-like6 | Glyma.05G124900 | 1644 | 547 | 6.16 | 61 |
| GmBBE-like7 | Glyma.05G125000 | 1611 | 536 | 9.25 | 61 |
| GmBBE-like8 | Glyma.05G125200 | 1593 | 530 | 8.75 | 59 |
| GmBBE-like9 | Glyma.05G125600 | 1749 | 582 | 6.48 | 64 |
| GmBBE-like10 | Glyma.05G125800 | 1596 | 531 | 9.09 | 60 |
| GmBBE-like11 | Glyma.05G125900 | 1605 | 534 | 9.63 | 60 |
| GmBBE-like12 | Glyma.06G321500 | 1584 | 527 | 8.91 | 60 |
| GmBBE-like13 | Glyma.06G321600 | 1590 | 529 | 7.68 | 60 |
| GmBBE-like14 | Glyma.08G059000 | 1593 | 530 | 8.87 | 59 |
| GmBBE-like15 | Glyma.08G059100 | 1596 | 531 | 8.38 | 60 |
| GmBBE-like16 | Glyma.08G079900 | 1620 | 539 | 7.64 | 61 |
| GmBBE-like17 | Glyma.08G080000 | 1584 | 527 | 8.43 | 58 |
| GmBBE-like18 | Glyma.08G080100 | 1590 | 529 | 9.13 | 59 |
| GmBBE-like19 | Glyma.08G080200 | 1596 | 531 | 8.36 | 60 |
| GmBBE-like20 | Glyma.08G080400 | 1734 | 577 | 6.85 | 64 |
| GmBBE-like21 | Glyma.08G080500 | 1620 | 539 | 8.57 | 60 |
| GmBBE-like22 | Glyma.08G080600 | 1584 | 527 | 8.81 | 59 |
| GmBBE-like23 | Glyma.08G080700 | 1572 | 523 | 9.57 | 58 |
| GmBBE-like24 | Glyma.08G080900 | 1782 | 593 | 9.67 | 67 |
| GmBBE-like25 | Glyma.08G112600 | 1608 | 535 | 6.25 | 60 |
| GmBBE-like26 | Glyma.09G022600 | 1602 | 533 | 9.68 | 60 |
| GmBBE-like27 | Glyma.09G026900 | 1632 | 543 | 6.58 | 61 |
| GmBBE-like28 | Glyma.09G027000 | 1647 | 548 | 9.59 | 61 |
| GmBBE-like29 | Glyma.09G027200 | 1602 | 533 | 9.22 | 59 |
| GmBBE-like30 | Glyma.09G027300 | 1602 | 533 | 8.75 | 59 |
| GmBBE-like31 | Glyma.09G028100 | 1698 | 565 | 8.68 | 64 |
| GmBBE-like32 | Glyma.09G028200 | 1332 | 443 | 9.45 | 50 |
| GmBBE-like33 | Glyma.09G028300 | 1614 | 537 | 9.18 | 60 |
| GmBBE-like34 | Glyma.10G177100 | 1653 | 550 | 5.73 | 61 |
| GmBBE-like35 | Glyma.15G132600 | 1632 | 543 | 8.04 | 61 |
| GmBBE-like36 | Glyma.15G132700 | 1569 | 522 | 8.48 | 58 |
| GmBBE-like37 | Glyma.15G132800 | 1635 | 544 | 9.02 | 61 |
| GmBBE-like38 | Glyma.15G132900 | 1614 | 537 | 6.52 | 60 |
| GmBBE-like39 | Glyma.15G133100 | 1536 | 511 | 8.61 | 57 |
| GmBBE-like40 | Glyma.15G133200 | 1479 | 492 | 9.68 | 55 |
| GmBBE-like41 | Glyma.15G133900 | 1680 | 559 | 8.61 | 63 |
| GmBBE-like42 | Glyma.15G134200 | 1626 | 541 | 9.56 | 61 |
| GmBBE-like43 | Glyma.15G134300 | 1608 | 535 | 9.17 | 60 |
| GmBBE-like44 | Glyma.15G153700 | 1452 | 483 | 8.22 | 55 |
| GmBBE-like45 | Glyma.20G213100 | 1653 | 550 | 5.54 | 61 |
